# An integrated approach for the in vitro dosimetry of engineered nanomaterials

**DOI:** 10.1186/1743-8977-11-20

**Published:** 2014-05-01

**Authors:** Joel M Cohen, Justin G Teeguarden, Philip Demokritou

**Affiliations:** 1 Center for Nanotechnology and Nanotoxicology, Department of Environmental Health, Harvard School of Public Health, Boston, MA, USA; 2 Systems Toxicology and Exposure Science, Pacific Northwest National Laboratory, Richland, WA, USA

## Abstract

**Background:**

There is a great need for screening tools capable of rapidly assessing nanomaterial toxicity. One impediment to the development of reliable *in vitro* screening methods is the need for accurate measures of cellular dose. We present here a methodology that enables accurate determination of delivered to cell dose metrics. This methodology includes (1) standardization of engineered nanomaterial (ENM) suspension preparation; (2) measurement of ENM characteristics controlling delivery to cells in culture; and (3) calculation of delivered dose as a function of exposure time using the ISDD model. The approach is validated against experimentally measured doses, and simplified analytical expressions for the delivered dose (Relevant In Vitro Dose (RID)_f_ function) are derived for 20 ENMs. These functions can be used by nanotoxicologists to accurately calculate the total mass (RID_M_), surface area (RID_SA_), or particle number (RID_N_) delivered to cells as a function of exposure time.

**Results:**

The proposed methodology was used to derive the effective density, agglomerate diameter and RID functions for 17 industrially-relevant metal and metal oxide ENMs, two carbonaceous nanoparticles, and non-agglomerating gold nanospheres, for two well plate configurations (96 and 384 well plates). For agglomerating ENMs, the measured effective density was on average 60% below the material density. We report great variability in delivered dose metrics, with some materials depositing within 24 hours while others require over 100 hours for delivery to cells. A neutron-activated tracer particle system was employed to validate the proposed *in vitro* dosimetry methodology for a number of ENMs (measured delivered to cell dose within 9% of estimated).

**Conclusions:**

Our findings confirm and extend experimental and computational evidence that agglomerate characteristics affect the dose delivered to cells. Therefore measurement of these characteristics is critical for effective use of *in vitro* systems for nanotoxicology. The mixed experimental/computational approach to cellular dosimetry proposed and validated here can be used by nanotoxicologists to accurately calculate the delivered to cell dose metrics for various ENMs and *in vitro* conditions as a function of exposure time. The RID functions and characterization data for widely used ENMs presented here can together be used by experimentalists to design and interpret toxicity studies.

## Introduction

Growing evidence suggests human exposure to engineered nanomaterials (ENMs) are inevitable [1,2] and may lead to adverse health effects where exposures are high enough [3-7], though the underlying toxicity mechanisms are not currently well-understood [8,9]. There is therefore great need for efficient and cost-effective toxicological screening to keep apace of the rapidly growing array of ENMs entering the consumer market [10-14]. Given the high cost of animal testing, reliable high-throughput *in vitro* screening methods are an attractive option for quickly and inexpensively characterizing the relationships between ENM physicochemical properties including size, morphology, surface chemistry, and crystallinity, and their biological effects [12,13,15,16].

However, to date *in vitro* assays have produced conflicting results that often disagree with animal data [6,10,17-19]. One impediment to the development of reliable *in vitro* screening methods is the need for accurate dosimetry [10,15-18]. Nanotoxicologists often report *in vitro* exposure doses in terms of administered mass or mass concentration, though scientific evidence continues to grow associating ENM toxicity with other dose metrics such as chemical reactivity due to total surface area or total particle number [16,20-22]. More importantly, the use of administered or nominal concentrations of particles in these systems ignores important processes (diffusion and sedimentation) that define their fate and transport and the rate of delivery to cells. These processes are strongly influenced by particle and media characteristics.

In a typical *in vitro* cytotoxicity study, ENM powders are usually suspended in liquid media for application to cells. Once suspended in liquid, ENMs often form large fractal agglomerates [23-26] thereby altering (1) the total number of free particles, (2) the total surface area available for biointeractions, and (3) the effective size and density of the particles [24,27]. Nanoparticle agglomerates are porous, containing media trapped during formation, with an “effective density” which is less than the density of the primary particles [23,28,29]. DeLoid et al. recently reported the broadest assessment to date, showing that the effective density for many flame-generated fractal ENMs in culture media was significantly lower than the material density [24], Notably, in contrast to soluble chemicals as well as their micron-sized counterparts, nanoparticle agglomerates can settle and diffuse differentially according to their hydrodynamic diameter and effective density, processes that are expected to significantly affect the delivered cellular dose as a function of exposure time [23,29-33].

Until recently, this phenomena had been demonstrated and quantified experimentally for a very limited number of nanoparticles [30,33-35], although Cohen et al. confirmed the generalizability of these results to a much broader group of materials by simulation [23]. For example, for some commonly used ENMs such as SiO_2_ suspended in culture media (hydrodynamic diameter: 227 nm; effective density:1.147 g/cm^3^), Cohen et al. estimated delivery of the entire administered dose to cells in culture can take up to hundreds of hours, indicative of the great importance of ENM interactions in physiological fluid and their subsequent effect on particle delivery to cells [23,24]. More importantly, the lack of key experimental methods for measuring the effective density of agglomerates presented a major obstacle for considering *in vitro* dosimetry in nanotoxicology studies, a challenge recently overcome by our group [24].

The recently developed Harvard Volumetric Centrifugation Method (VCM) is a simple, user- friendly method for experimentally determining the effective density of agglomerates under the conditions of study for *in vitro* systems. The accuracy of the VCM has been validated by comparison to established, high accuracy, but more costly and time consuming methods [24,27]. With the advent of the Harvard VCM, consideration of accurate dosimetry by *in vitro* nanotoxicologists in an fast and cost effective manner is now possible. Coupled with existing dosimetry algorithms such as the In vitro Sedimentation, Diffusion and Dosimetry model (ISDD) [23,27,29] particle and media properties along with the measured effective density can be used to calculate the number, mass and surface area of particles delivered to cell dose as a function of exposure time. The new approach significantly improves the accuracy and validity of high throughput *in vitro* toxicity screens currently employed in nanotoxicology studies, which are based on administered to cell dose [36,37]. It must be noted that due to the many assumptions of spherical agglomerates in the estimation of particle fate and transport via diffusion and sedimentation, the mobility of high aspect ratio materials may not be so easily estimated, and our group is currently working to characterize the mobility of such materials and determine whether the proposed approach may be applicable.

We present here an integrated methodology for *in vitro* particle dosimetry that takes into account the particokinetics in an *in vitro* system and enables accurate determination and reporting of delivered to cell dose metrics. Simple mathematical equations, referred to as Relevant In Vitro Dose (RID)_f_ functions that represent the rate of delivery of each particle under the given experimental conditions as a function of exposure time, are derived based on the proposed methodology for twenty widely used ENMs and are ready for use by toxicologists. The term “relevant” here refers to the dose actually delivered to cells, rather than the typically reported administered dose of particles in suspension. The reported RID_f_ functions can be used by *in vitro* nanotoxicologists to accurately calculate the particle mass (RID_M_), particle surface area (RID_SA_), or particle number (RID_N_) delivered to cells as a function of exposure time.

## Results and discussion

### ENMs investigated

Seventeen metal oxide ENMs covering representative oxides across the periodic table, two low aspect ratio carbonaceous nanoparticles, and non-agglomerating gold nanospheres typically used for biomedical applications were selected for study. Several of the materials were synthesized in house by flame spray pyrolysis (SiO_2_, CeO_2_, Fe_2_O_3_, Fe_3_O_4_, and TiO_2_) using the Harvard VENGES generation system [7,38,39]. It is worth noting that flame generated ENMs represent an industry-relevant class of materials which comprises 90% by volume of ENMs in the market [40]. The remaining materials were purchased from commercial sources, and their physico-chemical and morphological properties have been previously reported in great detail [37]. Primary particle size as determined by BET or transmission electron microscopy (TEM) method for all ENMs is summarized in Table 1. In general, primary particle sizes were in the range of 10-100 nm.

**Table 1:** Delivered dose metrics

**Material primary**	**Primary particle size (nm)**	**ρ**_**ENM**_**(g/cm**^**3**^**)**	**ρ**_**EV**_**(g/cm**^**3**^**)**	**d**_**H**_**(nm)**	**96 well plate**		**384 well plate**	
					**α (h**^**−1**^**)**	**t**_**90**_**(h)**	**α (h**^**−1**^**)**	**t**_**90**_**(h)**
Al_2_O_3_	14.7	3.97	1.81	57	0.0213	108	0.0317	72.7
CeO_2_	18.3	7.22	1.43	416	0.116	19.8	0.166	13.9
CoO	71.8	6.44	2.56	199	0.969	23.7	0.142	16.1
Cr_2_O_3_	193	5.22	2.21	358	0.246	9.37	0.343	6.71
Fe_2_O_3_	12.3	5.25	1.91	310	0.140	16.5	0.197	11.7
Fe_3_O_4_	12	5.17	1.46	274	0.0470	49.0	0.0751	30.7
Gd_2_O_3_	43.8	7.41	1.57	296	0.0730	31.5	0.112	20.6
Mn_2_O_3_	28.4	5.00	1.81	304	0.118	19.4	0.169	13.6
SiO2	13.5	2.65	1.30	40	0.0259	88.9	0.0406	56.7
TiO_2_	12.6	3.90	1.36	276	0.0366	62.9	0.0582	39.6
ZrO_2_	40.1	5.68	2.53	226	0.124	18.5	0.177	13.0
Printex (carbon black)	4.16	1.25	1.04	247	0.0126	183	0.0169	136
Carbon Nanohorns	20.3	1.25	1.02	261	0.0101	227	0.0139	166
VENGES SiO_2_	18.6	2.65	1.11	136	0.0138	166	0.0195	118
VENGES Fe_2_O_3_	27.6	5.245	1.52	380	0.117	19.6	0.167	13.8
VENGES CeO_2_-A	5.4	7.21	1.47	179	0.0288	80.0	0.0444	51.8
VENGES CeO_2_-B	27.9	7.21	1.62	181	0.0339	67.9	0.0525	43.8
VENGES CeO_2_-C	71.3	7.21	2.37	215	0.0991	23.2	0.145	15.9
EVONIK SiO_2_	14	2.65	1.15	227	0.0203	113	0.0297	77.6
Au Nanospheres	20*	19.3	17.7	42.2	0.0747	30.8	0.117	19.7

ρ_ENM_: material density (g/cm^3^); ρ_EV_: agglomerate effective density (g/cm^3^); d_H_: hydrodynamic diameter (nm); α: deposition fraction constant, (h^−1^); and t_90_: time for delivery of 90% of administered dose (h), for each ENM-media-concentration-well plate combination. Appropriate α can be used to accurately estimate the fraction of administered dose delivered to cells for any given exposure duration (see Equation 10).

### Characterization of ENM agglomerates in physiological media

Hydrodynamic diameter measured by DLS for all ENMs suspended at a concentration typically used for in vitro study (50 μg/ml) in RPMI tissue culture media supplemented with 10% fetal bovine serum (RPMI/10%FBS) is reported in Table 1. This data highlights the material specific effects on agglomerate size, and in general ENM formed agglomerates that were below 500 nm in diameter. This is in agreement with similar data reported in the literature for these ENMs and media conditions [23,24,37].

The measured agglomerate effective density for each ENM suspended in RPMI media was determined by the previously established Harvard Volumetric Centrifugation Method (VCM), and are also summarized in Table 1 (see Methods for details).

As expected, due to the fractal nature of flame-generated metal oxide ENMs, these materials exhibited effective density (ρ_EV_) values significantly lower than that of their material density (ρ_ENM_). This is a clear indication of the formation of porous agglomerates containing large amounts of trapped intra-agglomerate media. Non-agglomerating gold nanospheres exhibited a *ρ*_*EV*_ value only slightly less than the density of elemental gold (17.73 vs 19.3 g/cm3), consistent with minimal agglomeration, as expected. In general *ρ*_*EV*_ for all ENMs investigated correlated with raw material density, which may suggest that agglomerates of these ENMs are composed of comparable relative proportions of raw ENM and trapped media. For example, the lowest effective densities were measured for particles with the lowest material densities (Carbon nanohorns *ρ*_*EV*_ = 1.022 g/cm^3^, *ρ*_*ENM*_ = 1.25 g/cm^3^; Printex *ρ*_*EV*_ = 1.039 g/cm^3^, *ρ*_*ENM*_ = 1.25 g/cm^3^; VENGES SiO_2_*ρ*_*EV*_ = 1.112 g/cm^3^, *ρ*_*ENM*_ = 2.648 g/cm^3^), and the highest effective densities were measured for particles with the material densities greater than 5 g/cm^3^ (ZrO_2_*ρ*_*EV*_ = 2.528 g/cm^3^, *ρ*_*ENM*_ = 5.68 g/cm^3^; CoO *ρ*_*EV*_ = 2.56 g/cm^3^, *ρ*_*ENM*_ = 6.44 g/cm^3^; Au *ρ*_*EV*_ = 17.73 g/cm^3^, *ρ*_*ENM*_ = 19.3 g/cm^3^).

It is worth noting here that the reported values in this study for agglomerate diameter and effective density constitutes an extensive material property library reported for the first time in the literature which can be used by nanotoxicologists for dosimetry calculations for similar material-media-concentration combinations.

### Delivered to cell dose metrics

Great emphasis has been placed on determining the most appropriate dose metric for *in vitro* toxicity, be it mass, particle number, or surface area [21,32,41]. Importantly, as shown above, ENMs in suspension can form large agglomerates close to ten times their primary particle diameter, thereby altering the total number of free particles in suspension as well as the total surface area available for biointeractions before they even reach the cells cultured *in vitro*. Therefore, considerations of particle number and surface area dose metrics should take into account ENM transformations in liquid as described below.

For an ENM suspension of known mass concentration, *γ* (μg/ml), the total mass dose, *M* (μg), can be calculated as:

(1) M=γ×V

where *V* is the volume of exposure media (ml) applied directly to the cells in culture.

The total particle number dose, *N* (*#*), can be calculated from the total mass, *M*, hydrodynamic radius, *r*_*H*_ (cm, determined by DLS for ENMs in suspension), and agglomerate effective density, *ρ*_*E*_, (g/cm^3^), assuming spherical agglomerates, as:

(2) $$N=\frac{M}{\left(\frac{4}{3}\pi r_{H}^{3}\right)\times \rho_{E}}$$

Total surface area dose, *SA* (cm^2^), can then be calculated assuming spherical agglomerates as:

(3) $$\mathrm{SA}=\left(4\pi r_{H}^{2}\right)\times N$$

It is worth noting that cellular response to a biologically active material reflects the quantity of the substance actually coming into contact with the cells. For example, Wittmaack recently reported significant correlation between *in vitro* toxicity of SiO_2_ nanoparticles and the areal density of nanoparticle mass delivered to cells over the exposure duration [41]. Sharma et al. report similar findings for agglomerating iron oxide nanoparticles [35]. Cellular toxicity *in vitro* is therefore more accurately represented in relation to the delivered dose for any selected dose metric, rather than the typically reported administered mass concentration of ENMs in suspension.

### Deriving the Relevant In Vitro Dosimetry (RID) functions

Using the recently characterized material-media specific parameters for agglomerate hydrodynamic diameter and effective density (see Table 1) as inputs to any fate and transport algorithms, the fraction of administered particles that would deposit onto cells as a function of time, f(t), can be calculated. Alternatively, delivered doses to cells and fractional deposition can be directly measured as a function of time. In this study, the recently developed ISDD model was used to calculate the f(t) fractions using effective agglomerate densities measured using the Harvard VCM. It is worth noting that previous applications of ISDD were limited by the need to estimate agglomerate effective density using the Sterling Equation and assumptions about the fractal nature of the agglomerates. In addition to some uncertainty imposed by these assumptions, differences between actual and simulated doses can result [23,24,29]. Here we used the effective density directly measured by VCM for our dosimetry calculations.

The estimated f(t) function was then fitted as a Gompertz sigmoidal function as previously described by the authors [23] as follows:

(4) $$f_{D}\left(t\right)=1-e^{-\mathrm{\alpha t}}$$

Where α (hrs^−1^) is the material-media specific deposition fraction constant, and t (hr) is exposure duration.

The Relevant *In Vitro* Dose (RID)_f_ functions for delivered mass, number and surface area as a function of time can be derived by combining Equation 4 with Equations 1, 2 and 3 as follows:

For particle mass delivered to cells (RID_M_, μg):

(5) $$\mathrm{RI}D_{M}=\left(1-e^{-\mathrm{\alpha t}}\right)\times M$$

For particle number delivered to cells (RID_N_, number of particles).

(6) $$\mathrm{RI}D_{N}=\left(1-e^{-\mathrm{\alpha t}}\right)\times N$$

For total particle surface area delivered to cells (RID_SA_, cm^2^),

(7) $$\mathrm{RI}D_{\mathrm{SA}}=\left(1-e^{-\mathrm{\alpha t}}\right)\times \mathrm{SA}$$

While validated computational models such as the ISDD model are readily available to nanotoxicologists to perform accurate estimates for delivered dose as described above, the RID functions presented here constitute an inherent property of the ENM, media and well plate *in vitro* system. RID functions derived for these specific conditions can be used to easily and accurately calculate delivered to cell dose metrics, as a function of time, without the use of sophisticated fate and transport numerical algorithms.

RID functions are provided here for all twenty ENMs and conditions used in this study. The necessary parameter values, consisting of the deposition fraction constant (α), agglomerate size (r_H_), and agglomerate effective density (ρ_e_) are reported in Table 1 for various ENMs and well plate geometries used in this study. However the aforementioned methodology can be followed for any ENM, media and plate configuration to derive the specific RID function for any other material, media and geometry condition.

**Figure 1:**
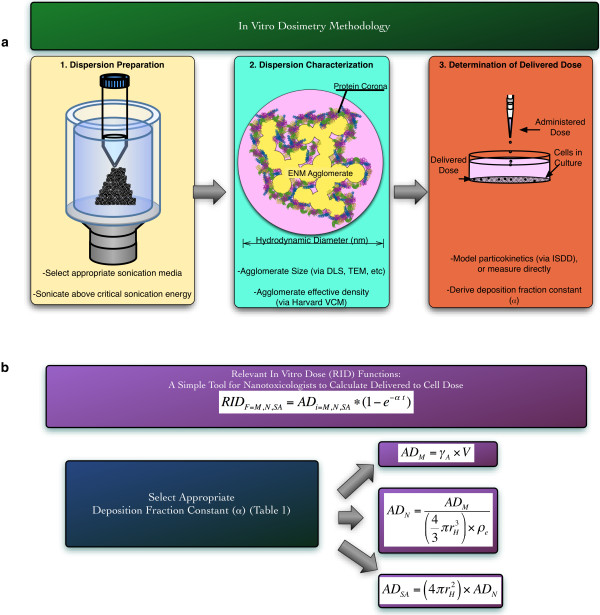
**Schematic Map for proposed integrated In Vitro Dosimetry Methodology. a**, Proper dispersion preparation requires selection of appropriate sonication media (such as DI H_2_O for metal oxide ENMs), and sonication above the critical sonication energy required to break ENMs down to the smallest possible agglomerates that are stable over time. Characterization of dispersion characteristics including agglomerate diameter and agglomerate effective density allow for accurate modeling of particokinetics in vitro, and determination of delivered dose metrics and the deposition fraction constant. **b**, Relevant In Vitro Dose functions (RID_f_) provide a simplified tool for nanotoxicologists to quickly estimate delivered dose values for the ENMs investigated in this manuscript. Selection of the appropriate deposition fraction constant (α, listed in Table 1), allows nanotoxicologists to directly calculate relevant in vitro doses (RID) for any exposure duration, including delivered ENM mass (RID_M_, μg), delivered particle number (RID_N_, #), and delivered surface area (RID_SA_, cm^2^), using the equations listed below. t is exposure duration (h), γ is ENM mass concentration (μg/ml), V is media volume applied to cells (ml), r_h_ is hydrodynamic radius (cm, listed in Table 1), and ρ_E_ is agglomerate effective density (g/cm^3^, listed in Table 1).

Figure 1a provides a schematic map of the proposed methodology to derive the RID functions for any ENM and *in vitro* conditions.

It is necessary to note that for materials that undergo significant dissolution over the course of the study, changes in agglomerate diameter and effective density that result from mass loss due to dissolution must be resolved over time and addressed in the fate and transport algorithm in order to accurately estimate delivered dose [24]. A recent dissolution study of 24 industrially relevant metal oxide nanoparticles incubated in cell culture media over 24 hours reported that very few particles exhibit greater than 10% mass loss due to dissolution (relatively highly soluble materials: ZnO, CuO, WO_3_) [37]. To ensure greatest accuracy for these materials, mass loss due to dissolution, *d*_*H*_, and *ρ*_*EV*_, should be measured over the time of exposure. These time- resolved values should then be utilized by the transport simulation model to accurately estimate delivered dose. Considering mass loss due to dissolution will likely result in a net decrease in effective density, any dosimetry calculations for soluble materials based on effective density measured immediately after sample preparation would provide an overestimate of particle deposition over time. In addition, for completion of dosimetry calculations, both the particulate and soluble components must be correctly identified and considered separately as previously described [24]. The ISDD model used here in its current form does not take into consideration time resolved data for effective density and hydrodynamic diameter.

### Experimental validation of the proposed integrated in vitro dosimetry methodology

**Figure 2:**
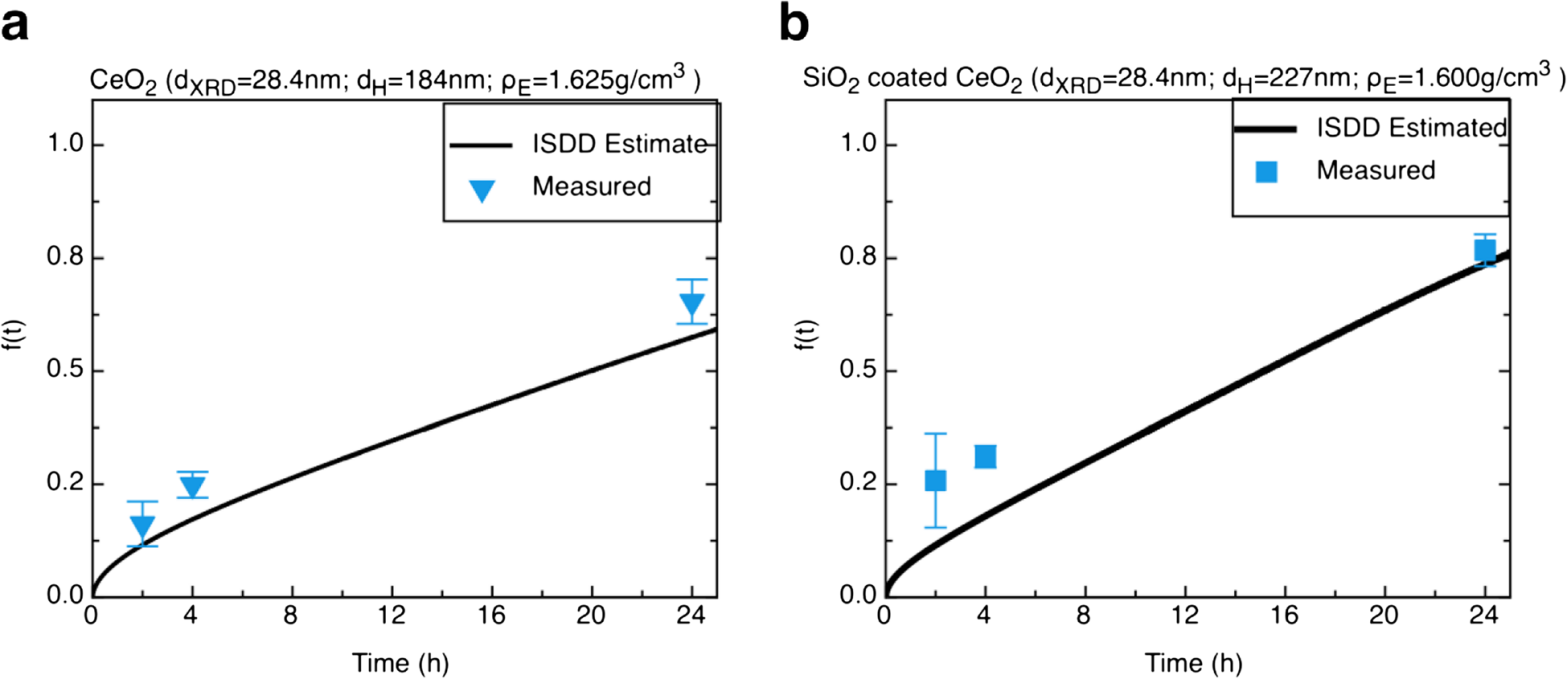
**Validation of dosimetry methodology for two metal oxide ENMs. a**. Validation of dosimetry approach for CeO_2_ (d_XRD_=28.4 nm) suspended in DMEM; **b**. Validation of dosimetry approach for SiO_2_ coated CeO_2_ (d_XRD_=28.4 nm) suspended in DMEM. All experiments were done in triplicate, error bars represent standard deviation.

In order to validate the proposed methodology, suspensions of two neutron-activated ENMs (CeO_2_, and SiO_2_ coated CeO_2_) were applied to transwell insert membranes with 3 μm pores (Additional file 1: Figure S1) as described in great detail in methods. Neutron activation of ENMs has been used routinely in our lab for biokinetic and in vitro studies of ENMs [42]. This is a well-established method for accurately tracking the gamma emitting isotope ^141^Ce with high sensitivity and correlates extremely well with total particle mass [43]. Following 2, 4, and 24 hours incubation we measured the delivered dose by gamma spectroscopy, defined as the sum of particles that deposited on or passed through membrane. For both materials, less than 2% of the administered dose remained stuck to the membrane suggesting easy passage of ENMs through the 3 μm pores with minimum particle losses (data not shown). Additionally, the delivered dose was estimated using the proposed *in vitro* dosimetry methodology presented here. As shown in Figure 2, there is a close agreement between the measured delivered dose and the estimated delivered dose for both materials at each time point. This is a clear validation of the proposed dosimetric approach for ENMs.

It is worth noting that the dosimetry model (ISDD) used in this study as the fate and transport algorithm in deriving the RID functions has been validated for a limited number of materials including non-agglomerating fluorescently labeled polystyrene beads of various agglomerate diameter [29,34], and for super paramagnetic iron oxide particles [29], using only estimates of effective density. The data presented here with the neutron activation tracer particle approach extends that previous validation to two additional flame-generated and industrially-relevant materials. Additionally, the validation reported here improves upon major uncertainties of the previous work by employing direct measurements of agglomerate density by the Harvard VCM for industry relevant materials.

### Utility of the proposed RID functions to estimate in vitro dosimetry of ENMs

The simple mathematical RID functions obtained here for the extensive panel of ENMs can be used by nanotoxicologists to accurately estimate the delivered to cell dose metrics (mass, particle number, total surface) as a function of time. The material and well geometry specific deposition fraction constant, α (h^−1^), and the time required to deliver 90% of the administered dose, t_90_ (h) for each ENM-media-concentration system are summarized in Table 1.

#### Impact of effective density and hydrodynamic diameter on dosimetry

In general, ENMs having greater values for both effective density and agglomerate diameter were expected to deposit more rapidly than those with smaller values for both properties. For example, Cr_2_O_3_ exhibited the highest alpha parameter and shortest t_90_, consistent with its relatively large agglomerate diameter and effective density (d_H_ = 358 nm, ρ_EV_ = 2.21 g/cm^3^, α = 0.343, t_90_ = 6.71 h for a 384 well plate). In contrast, VENGES SiO_2_ exhibited a low alpha parameter and long t_90_, consistent with its relatively small agglomerate diameter and effective density (d_H_ = 136 nm, ρ_EV_ = 1.112 g/cm^3^, α = 0.0169, t_90_ = 118 h for a 384 well plate). This trend is consistent with previous reports in the literature of measured target dose *in vitro*[35].

**Figure 3:**
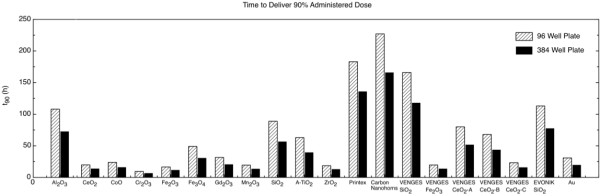
**Comparison of time required to deliver 90% of the administered dose (t**_**90**_**) in hours (h), calculated following the described dosimetry methodology, for all materials investigated in two well plate configurations.**

Figure 3 presents the time required to deliver 90% of the administered dose (t_90_), in hours (h), for all materials investigated, in two well plate geometries (96 and 384 well plate). These results confirm and significantly extend evidence that that the dose rates and target cell doses can vary significantly depending on the material, media, and well plate properties, and emphasize the importance of characterizing ENM-suspensions for both agglomerate diameter and effective density. For the materials investigated in this study, the time required to deliver particles to cells *in vitro* can differ by a factor of 20, and can range from <10 hours up to >100 hours (Figure 3). Consideration of the variability of delivered dose may hold large implications for the interpretation of previously reported high-throughput toxicity screens of large panels of ENMs [36,37].

Differences in delivered dose values provide a major challenge for comparisons of the dose–response curves between materials exhibiting fast transport (via sedimentation or diffusion), and materials exhibiting slow transport that achieve significantly lower delivered doses *in vitro*. These challenges can be partially addressed by reporting delivered doses directly measured *in vitro*, delivered doses estimated following our described methodology and the Harvard VCM, or delivered doses based on the reported RID functions reported in Table 1, rather than using the administered dose metric previously used in the study.

#### Future work

It is necessary to note that the reported dosimetry data for all ENMs was estimated based on agglomerate size and density measured immediately following sample preparation, assuming no agglomerate changes over time due to dissolution. The dissolution of a large panel of metal and metal oxide ENMs (many of which are included in this study) suspended in cell culture media following 24 hour incubation has been previously reported [37], and only two materials exhibit ≥10% dissolution (ZnO, and CuO). For partially soluble materials that may produce suspensions in which agglomerate size and effective density change over time, these properties must be resolved over time and the time-resolved values included in the fate and transport algorithm (e.g. ISDD), in order to accurately estimate delivered dose.

One potential limitation to the proposed dosimetry approach is the lack of validation for high aspect ratio nanomaterials such as carbon nanotubes. Considering the many assumptions of spherical agglomerates in the estimation of particle fate and transport via diffusion and sedimentation, the mobility of high aspect ratio materials may not be so easily estimated. Carbon nanotubes can form large irregularly shaped agglomerates in liquid suspension, the mobility of which may in fact be accurately estimated from the agglomerate diameter and effective density. Our group is currently working to characterize the mobility of such materials and determine whether the proposed approach may be applicable.

It must also be noted that agglomerate diameter may be greatly influenced by the mass concentration of particles in solution. The RID functions derived in this study were generally valid for a mass concentration range of 0-50 μg/ml (data not shown), which is within the usual levels used for *in vitro* studies [6,39]. Concentration-dependent effects on ENM agglomeration state occur for some of the tested ENMs at concentrations greater than 50 μg/ml (data not shown), and for concentrations higher than this range, the proposed methodology needs to be applied in order to derive revised RID functions based on effective density and agglomerate size for those high concentration levels. Further investigations into the influence of particle concentration on agglomeration state are forthcoming.

The protein corona is another important factor influencing nanoparticle agglomeration, particle delivery to cells, and cellular uptake and trafficking [44-46]. Our group recently reported that the presence and concentration of proteins in the suspension media can influence formed agglomerate size and stability over time [23]. This topic was also investigated utilizing atomic force microscopy techniques to determine the influence of proteins on agglomeration potential and particle-particle interactions in liquid suspension [47]. Future-work characterizing ENM-protein interactions and the implications for agglomerate parameters and cellular toxicity are necessary for the field of *in vitro* nanotoxicology.

Examples from the recent literature demonstrate the significant impact of dosimetry on interpreting the results of various in vitro assays. These principles were first described in detail several years ago [23,29,31], and are consistent with basic principles of pharmacology and toxicology as well as the gold standard practice for chemical risk assessment. More recently, our group reported on the impact of particle settling on interpreting the relative abilities of various flame-generated nanomaterials to translocate across alveolar epithelial monolayers in vitro [42]. Furthermore, with regards to hazard ranking large panels of ENMs, a recent investigation into the toxicity of low aspect ratio ENMs reported ratios of slopes for delivered dose vs administered dose varying between 1.02 and 5.58, with the rank order of ENMs shifting notably for some ENMs when delivered dose was taken into account (Pal et al., submitted 2014). Furthermore, the development of reliable *in vitro* screening assays requires identification of equivalent doses between *in vitro* and *in vivo* systems. One proposed approach was recently reported that utilized the multiple-path particle dosimetry model (MPPD) to estimate the deposited and retained dose of ENMs in the alveolar regions of exposed animals. These doses were then compared with cellular responses measured at equivalent doses of ENMs delivered to cells in vitro [6,48], Teeguarden et al., submitted 2014]. Future studies are necessary to further determine the impact of dosimetry on the hazard ranking of large panels of ENMs in both cellular and whole animal systems, though preliminary results suggest particle delivery to cells plays a significant role in nano-bio interactions *in vitro* and affect hazard ranking for some ENMs and endpoints. More importantly, there is consensus in the nanotoxicology field that there is a need to bridge the gap between in vitro and in vivo models, which requires reporting of biological response data in vitro and in vivo on the same dose scale- the amount of material deposited to cells/tissue, rather than the administered mass dose metric currently used in nanotoxicology studies. The proposed dosimetric approach can be a valuable and easy to use tool for nanotoxicologists in their quest of understanding the toxicological implications of ENMs.

Direct measurement of the internalized cellular dose is another dose metric worth investigating to further elucidate the mechanisms of nanomaterial toxicity *in vitro*. Our group recently demonstrated for a small panel of neutron activated ENMs that particle uptake and trafficking is dependent on particle delivery to cells, and provided electron micrographs and DLS data characterizing the agglomeration state of internalized and exocytosed particles [42]. Future work is necessary to characterize cellular internalization of ENMs *in vitro*, and it will be useful to compare the dose response curves based on administered, delivered, and internalized doses.

## Conclusions

Our integrated *in vitro* dosimetry methodology and the derived RID functions provide a tool for nanotoxicologists to accurately calculate the particle mass (RID_M_), particle surface area (RID_SA_), or particle number (RID_N_) delivered to cells in culture as a function of exposure time. This methodology is based on direct measurement of agglomerate parameters, and is an improvement upon previous work relying on unvalidated estimates for agglomerate effective density [29]. Numerical calculations for particle deposition over time have been validated experimentally for a variety of materials and conditions, highlighting the accuracy and wide applicability of this method to industrially-relevant ENMs. Furthermore, the RID functions presented here for 20 ENMs in a variety of *in vitro* conditions can assist nanotoxicologists in addressing dosimetry issues in their *in vitro* screening studies. Adoption of the proposed dosimetry methodology will be a major step towards the development of inexpensive, accurate, and reproducible *in vitro* screening assays, and will overcome potential inaccuracies that may arise from reporting dose as simply the nominal media concentrations. Consideration of the variability of delivered dose may hold large implications for the interpretation of previously reported high-throughput toxicity screens of large panels of ENMs [36,37], and will be a major advancement for nano-environmental health and safety research in the future.

## Materials and methods

### Nanomaterials and characterization

ENMs investigated are listed in Table 1. SiO_2_, Fe2O_3_, and CeO_2_ ENM powders were generated in-house by flame spray pyrolysis using the Harvard Versatile Engineered Nanomaterial Generation System (VENGES) recently developed by the authors [7,38]. Additional metal oxide ENM powders were purchased from commercial vendors (SiO_2_ and TiO_2_: EVONIK, Essen, Germany; Al_2_O_3_, Cr_2_O_3_, Fe2O3, Mn_2_O_3_, and Zr_2_O_3_: US Research Nanoaterials Inc., Houston, TX; CoO: Skyspring Nanomaterials Inc., Houston, TX; Gd_2_O_3_ and SiO_2_: Nanostructured and Amorphous Materials Inc., Houston, TX; CeO_2_: Meliorum Technologies Inc., Rochester, NY). Carbon nanohorns and Printex-90 carbon black particles were donated by Dr. Dhimiter Bellow at University of Massachussetts Lowell, and characterized previously in the literature [49,50].

Spherical monodisperse gold nanospheres, which were donated by Dr. Srinivas Sridhar at Northeastern University, were prepared in suspension as previously described [51]. Briefly, 500 ml of 1 mM HAuCl_4_ in a round bottom flask was heated to a rolling boil with vigorous stirring. 50 ml of 38.8 mM sodium citrate solution was added rapidly. After a color change from pale yellow to purple, indicating formation of gold nanoparticles, boiling was continued for another 15 minutes, after which the heating source was removed and the suspension stirred for an additional 15 minutes. The suspension was filtered through 0.45 μm syringe filters and stored at 4°C.

For powdered ENMs specific surface area, *SSA*, defined as the particle surface area per mass (m^2^/g), was determined by the nitrogen adsorption/Brunauer-Emmett-Teller (BET) method using a Micrometrics Tristar 3000 (Micrometrics, Inc., Norcross, GA, USA) for each ENM. The equivalent primary particle diameter, *d*_*BET*_, was calculated, assuming spherical particles, as

(8) $$d_{\mathrm{BET}}=\frac{6}{\mathrm{SSA}\times \rho_{P}},$$

where *ρ*_*p*_ is the particle density, which was obtained for each particle from the densities of component materials, at 20°C, reported in the CRC handbook of Chemistry and Physics [52]. ENM powder primary particle morphology and size were further characterized, and for monodisperse gold nanospheres primary particle diameter was estimated by transmission electron microscopy (TEM) using a Zeiss Libra 120 microscope (Carl Zeiss GmbH, Jena, Germany).

### ENM dispersal and characterization in suspension

The material specific delivered sonication energy required to achieve stable monodisperse agglomerates in suspension (DSE_CR_) was determined following a previously established protocol [23,37]. In brief, ENMs were dispersed in deionized water at 5 mg/ml by probe sonication, calorimetric calibrated whereby the power delivered to the sample was determined to be 3 W. Suspensions are then characterized for hydrodynamic diameter (nm), polydispersity, zeta potential (mV), and specific conductance (mS/cm) by dynamic light scattering (DLS) using a Dynapro Plate Reader (Wyatt Technology) and ZetaPALS instrument (Zeta Potential Analyzer, Brookhaven Instruments Corporation, Holtsville, NY). Plots of hydrodynamic diameter as a function of DSE exhibiting asymptotic de-agglomeration trends are derived for each ENM and used to determine the material-specific DSE_CR_, corresponding to the lowest agglomeration state. From each curve, an ENM-specific DSE_CR_ is estimated by determining the DSE value at which the dispersed ENMs were within 10% of their observed minimum hydrodynamic diameter as measured by DLS. To determine the stability of the sonicated suspension over time, hydrodynamic diameter measurements were repeated for several hours following sonication.

### Effective density by volumetric centrifugation method (VCM)

One ml samples of 100 μg/ml suspensions of metal oxide ENMs were dispensed into TPP packed cell volume (PCV) tubes (Techno Plastic Products, Trasadingen, Switzerland) and centrifuged at 2,000 × *g* for one hour. Agglomerate pellet volumes, *V*_*pellet*_, were measured using a slide rule-like *easy-measure* device also obtained from the PCV tube manufacturer. Effective agglomerate densities were calculated from *V*_*pellet*_ values of triplicate samples for each ENM and condition as described previously [24]. Media density was calculated from the mass of a 50 ml sample by subtracting the weight of a 50 ml volumetric flask from the weight of the same flask containing 50 ml of media (RPMI supplemented with 10% fetal bovine serum, RPMI/10%FBS) at 20°C, and the theoretical stacking factor (SF) of 0.634 based on stacking of irregular spheres was used for all materials [53].

### Delivered dose computation

The *in vitro* sedimentation, diffusion and dosimetry (ISDD) model developed by Hinderliter *et al.*[29], was used to calculate the fraction of administered particles deposited standard 96- and 384-well plates as a function of time *f*_*D*_*(t)* as previously described [23,24]. In addition to effective density, ISDD model inputs included the hydrodynamic diameter, *d*_*H*_, measured by DLS in the test media, at 50 μg/ml, the media column height (3.16 mm for 100 μl exposure media in 96 well plates, 2.27 for 25 μl exposure media in 384 well plates), temperature (310 K), media density, (1.00 g/cm^3^), media dynamic viscosity (0.00074 Pa s) [29], and administered (initial suspension) particle concentrations of 50 μg/ml. For each ENM the model-derived *f*_D_ (t) was fit to a Gompertz sigmoidal equation,

(9) $$f_{D}\left(t\right)=1-e^{-\mathrm{\alpha t}},$$

where *t* is time (h), and *α* is an ENM- and media-specific deposition fraction constant (h^−1^). Solving Equation (9) for the time *t* at which the fraction *f*_*D*_*(t)* of administered particles is delivered yields

(10) $$t=-\frac{\ln\left(1‒f_{D}\left(t\right)\right)}{\alpha }.$$

Equation (10) was used to calculate the time required for delivery of 90% of the administered dose, *t*90, for each ENM dispersion using the specific deposition function constants, *α*, and an *f*D(t) value of 0.90.

### Validation of proposed dosimetry methodology using neutron activated ENMs

ENM powders were irradiated with neutrons for up to 24 hours at the Nuclear Reactor Laboratory (Massachusetts Institute of Technology, Cambridge, MA), and the radioactive ^141^Ce ENMs (CeO_2_, SiO_2_-coated CeO_2_) were stored in irradiation tubes. ^141^Ce is a gamma emitter with a half-life of 32 days, and the successful production of radioactive ENMs following irradiation was confirmed by gamma energy spectrometry using a Packard gamma counter (Cobra Quantum, Packard Instrument, IL). ENMs were quantified as either percentage of total administered mass dose, based on total measured radioactivity (counts per minute, CPM). Concentration calibrations were performed for each ENM by measuring radioactivity for a measured and known total ENM mass in suspension, and measuring radioactivity of dilutions by half down to 5 ng.

100 μl of ENM suspensions at a concentration of 12.5 μg/ml were applied to transwells without cells, for 2, 4, and 24 hours (see Additional file 1: Figure S1). Following exposure, all supernatant and transwell inserts were collected from culture plates and set aside for analysis by gamma spectroscopy. Supernatants were collected from the transwell, and transwell inserts were set aside for measurement by gamma spectroscopy. All liquid present in the basolateral compartment of the transwell was then collected, and each basal well was washed 3 times with PBS and collected. Gamma counts were measured for apical compartments (including supernatant and the transwell insert), as well as for the basal compartment (including collected media and PBS wash) by gamma spectroscopy. A mass balance of ENMs measured in the apical and basal compartments was compared to gamma readings for ENM suspensions of equivalent total particle mass (1.25 μg) to confirm minimal particle loss. All experiments were conducted in triplicate.

## Abbreviations

ENM: Engineered nanomaterials; VCM: Volumetric Centrifugation Method; RIDM: Relevant in vitro dose function for particle mass delivered to cells; RIDN: Relevant in vitro dose function for particle number delivered to cells; RIDSA: Relevant in vitro dose function for particle surface area delivered to cells; ISDD: In vitro sedimentation, diffusion and dosimetry model; TEM: Transmission electron microscopy; VENGES: Versatile Engineered Nanomaterial Generation System; BET: Brunauer-Emmett-Teller; DSECR: Critical delivered sonication energy; DLS: Dynamic light scattering; PCV: Packed cell volume; SF: Stacking factor; ρEV: Agglomerate effective density (g/cm^3^); ρENM: Material density (g/cm^3^); f(t): Fraction of administered particles deposited onto cells as a function of time; α: Material-media specific deposition fraction constant (hrs^−1^); t90: Time required to deliver 90% of the administered particle dose (h).

## Competing interests

None of the authors have any competing financial interests in the work described in this manuscript.

## Authors’ contributions

JMC co-developed the dosimetry methodology, carried out ENM suspension characterization, dosimetric modeling, and dosimetry validation experiments, and drafted the manuscript. JGT provided nanomaterials for characterization, provided the ISDD model, and drafted the manuscript. PD supervised the project, co-developed the dosimetry methodology, prepared VENGES nanomaterials, and drafted the manuscript. All authors read and approved the final manuscript.

## Supplementary Material

Additional file 1: Figure S1 Experimental setup for dosimetry methodology validation experiments. Click here for file
