# Supplementary material for: An integrated approach for the in vitro dosimetry of engineered nanomaterials
Source: Part Fibre Toxicol. 2014 May 1;11:20. doi: 10.1186/1743-8977-11-20 (PMC4024018; doi:10.1186/1743-8977-11-20)

**Additional file 1: Figure S1:** **Experimental setup for dosimetry methodology validation experiments.**


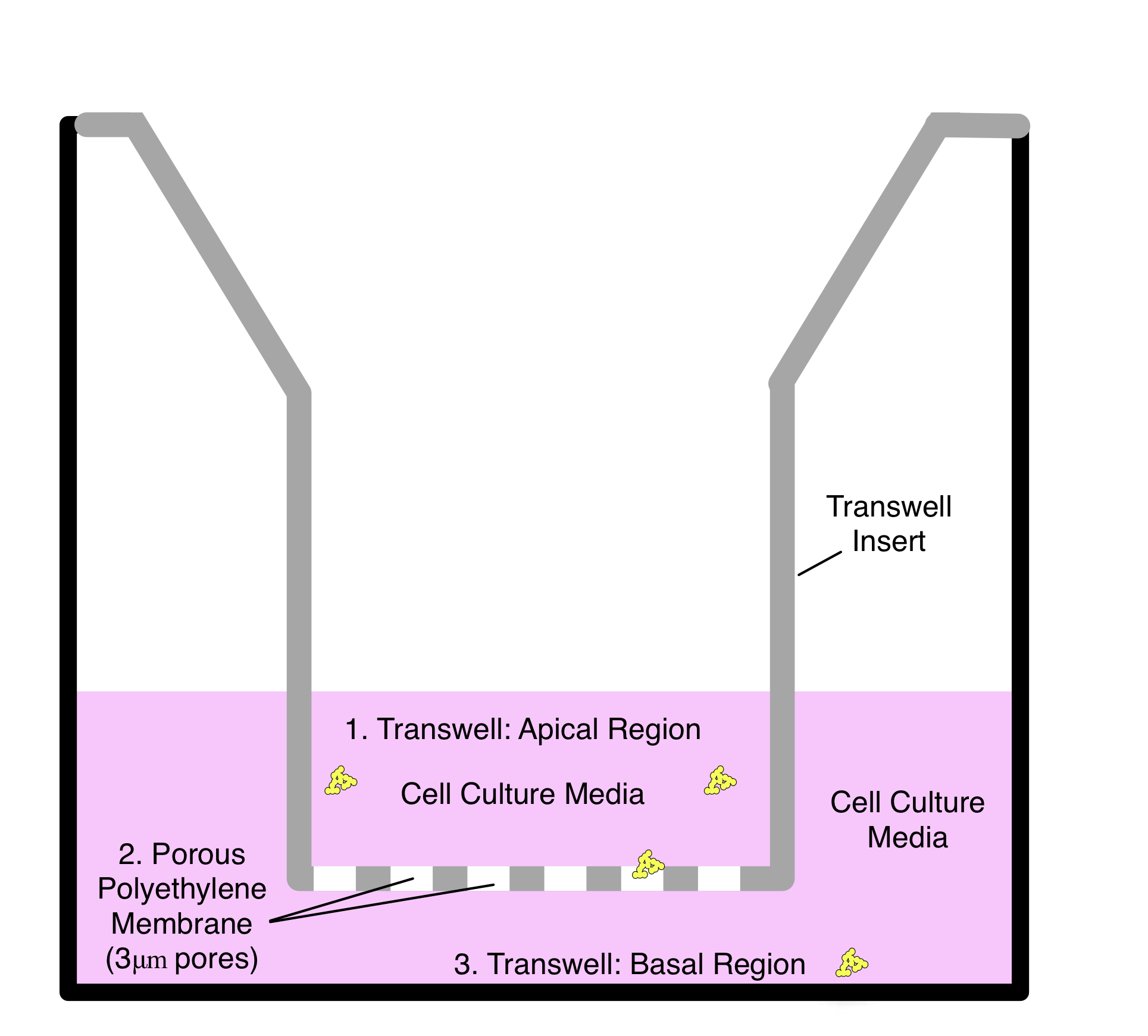

Supplement: Additional file 1: Figure S1 — Experimental setup for dosimetry methodology validation experiments. [file 1743-8977-11-20-S1.docx]
